# NMR metabolomics reveals effects of *Cryptosporidium* infections on host cell metabolome

**DOI:** 10.1186/s13099-019-0293-x

**Published:** 2019-04-03

**Authors:** Christopher N. Miller, Charalampos G. Panagos, William R. T. Mosedale, Martin Kváč, Mark J. Howard, Anastasios D. Tsaousis

**Affiliations:** 1 Laboratory of Molecular & Evolutionary Parasitology, RAPID Group, School of Biosciences, University of Kent, Canterbury, UK; 2 Biomolecular NMR Facility, School of Biosciences, University of Kent, Canterbury, UK; 3 Institute of Parasitology, Biology Centre CAS, Ceske Budejovice, Czech Republic; 4 Faculty of Agriculture, University of South Bohemia in České Budějovice, Ceske Budejovice, Czech Republic; 5 Present Address: Complex Carbohydrate Research Center, University of Georgia, Athens, GA 30602 USA; 6 Present Address: School of Chemistry, University of Leeds, Leeds, LS2 9JT UK

**Keywords:** Cryptosporidiosis, NMR, Metabolomics, COLO-680N, Taurine

## Abstract

**Background:**

*Cryptosporidium* is an important gut microbe whose contributions towards infant and immunocompromise patient mortality rates are steadily increasing. Over the last decade, we have seen the development of various tools and methods for studying *Cryptosporidium* infection and its interactions with their hosts. One area that is sorely overlooked is the effect infection has on host metabolic processes.

**Results:**

Using a ^1^H nuclear magnetic resonance approach to metabolomics, we have explored the nature of the mouse gut metabolome as well as providing the first insight into the metabolome of an infected cell line. Statistical analysis and predictive modelling demonstrated new understandings of the effects of a *Cryptosporidium* infection, while verifying the presence of known metabolic changes. Of note is the potential contribution of host derived taurine to the diarrhoeal aspects of the disease previously attributed to a solely parasite-based alteration of the gut environment, in addition to other metabolites involved with host cell catabolism.

**Conclusion:**

This approach will spearhead our understanding of the *Cryptosporidium*-host metabolic exchange and provide novel targets for tackling this deadly parasite.

**Electronic supplementary material:**

The online version of this article (10.1186/s13099-019-0293-x) contains supplementary material, which is available to authorized users.

## Background

Cryptosporidiosis is a disease characterised by prolonged episodes of intense diarrhoea and is the second largest cause of diarrheal disease and diarrhoea-associated deaths in infants across Africa and South Asia [1–4]. The aetiological agents of this disease are the apicomplexan parasites belonging to the *Cryptosporidium* genus. Cryptosporidiosis is also amongst one of the most common diseases of immunocompromised individuals, particularly HIV positive patients who are at 75–100% risk of contracting the disease during their lifetime [4, 5]. Nearly 20 *Cryptosporidium* species and genotypes are responsible for causing the disease in humans; although two species in particular, *Cryptosporidium hominis* and *C. parvum*, are most likely to be found in infected patients [2, 4, 6–9]. Infection occurs when an individual ingests the oocysts of the parasite, often by drinking a contaminated water source. Water treatment options are limited to filtering or boiling, which are generally not possible at an industrial scale and UV treatment, which is both expensive and rarely in place prior to outbreaks. Failing this, treatment is typically rehydration, although one drug has been shown to be effective, the broad spectrum anti-parasitic nitazoxanide [10]. The drug is far from ideal, however, and displays a range of undesirable side effects including cytotoxicity and nausea, as well as being limited to use in cases where the patients are immunocompetent [11–14].

Until recently, a significant barrier to research into cryptosporidiosis has been the absence of a combined long-term in vivo culturing system and comprehensive model of host parasite interactions in addition to a heavy reliance on antibody based detection both in the scientific and the medical field [1, 3, 15–19]. Recent papers have attempted to rectify this by proposing improved or entirely novel techniques for culturing the parasite ex vivo in cell cultures, using the cultured cancer cells as host cells [20, 21]. A recent study identified that infection of COLO-680N cell cultures produced a longer term and higher production volume culture of the parasite compared to previously existing in vitro cultures [22, 23]. These advances have allowed higher in depth microscopy-based studies and even promise to provide a solution to developing a genetic engineering platform for the parasite. However, beyond microscopy and localisation studies [24], the knowledgebase of the host parasite interaction remains largely undeveloped [3, 13, 14, 21, 25]. One area lacking study is metabolomics. The study of parasite metabolomics is becoming increasingly important as the search for preventative treatments and cures becomes increasingly specific [26]. However, before metabolomics can be used to satisfying effect, the tools must first be properly established and demonstrated.

To our knowledge, only two peer-reviewed publications have explored the concept of the infection metabolome, one on mice and the other on human faecal samples [27, 28]. The findings of each paper demonstrated a clear relationship between the infection and metabolic changes. Although working on different organisms and sampling sites, each study identified the hexadecanoic acid as a significant component of these changes. Other changes noticed included a decrease in the relative abundance of amino acids in infected mice faeces, although an increase was previously in humans [27]. This was explained to be most likely due to the inherent variation between the different host species metabolomes, as highlighted by Saric et al. in 2008 [29]. However, this highlights a pressing need for further and wider reaching studies into the metabolome of *Cryptosporidium* infections. One approach would be to increase the variety of tools available, in addition to the gas chromatography–mass spectrometry (GC–MS) used in those papers [27–29].

Currently, many metabolomics studies utilise a GC–MS approach, with great success, however ^1^H nuclear magnetic resonance (NMR) metabolomics can be used as an additional or alternative powerful tool for metabolic screening. ^1^H NMR is a simple method that allows for a comparatively lossless analysis of metabolites, with fewer steps between sample recovery and analysis than GC–MS, offering a huge advantage for studies involving time sensitive or limited resources restrictions, such field research [29–33]. This translates to a more reliable result in terms of quantification and immediate reproducibility. As such, NMR has already seen use in analysing the profile of *Plasmodium falciparum*, although the metabolome of the apicomplexan parasite, as is the case with the rest of the group, remains largely unexplored compared to similar studies of other organisms [34].

Here we investigated the host-parasite interactions, using a combination of microscopy and ^1^H NMR approaches. We validated our methodology by comparing faecal profile results to the previously published studies, which used different methodologies, namely GC–MS [27, 28]. Further experimentation utilised a recently published infectible culture, COLO-680N [22], to determine if any similarities or differences in response to infection could be determined.

## Results

### Cell culture sample extractions

**Fig. 1:**
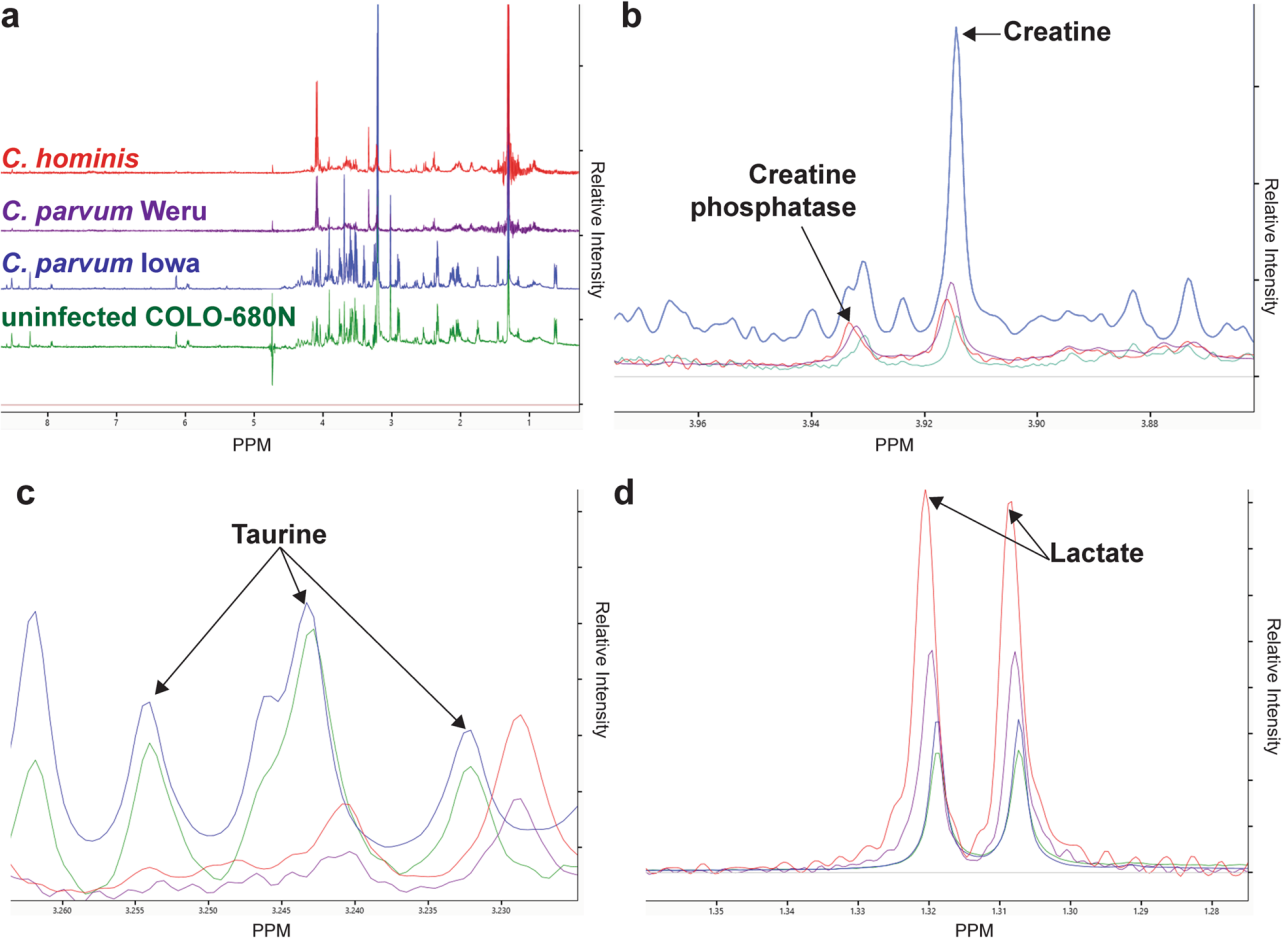
Cell Culture infection NMR spectra. **a** Stacked NMR Spectra produced from the COLO-680N control cultures (green), either the *C. parvum* Iowa II (blue), *C. parvum* Weru (purple), or *C. hominis* groups, 7 days’ post infection. Direct comparisons of the spectra revealed several clearly identifiably differences, including differences in creatine and creatine phosphate (**b**), taurine (**c**) and lactate (**d**) levels. Noticeably, taurine levels were almost undetectable in *C. hominis* or *C. parvum* Weru infections. The spectra displayed are of individual experiments and are representative of the spectra observed throughout the groups

**Fig. 2:**
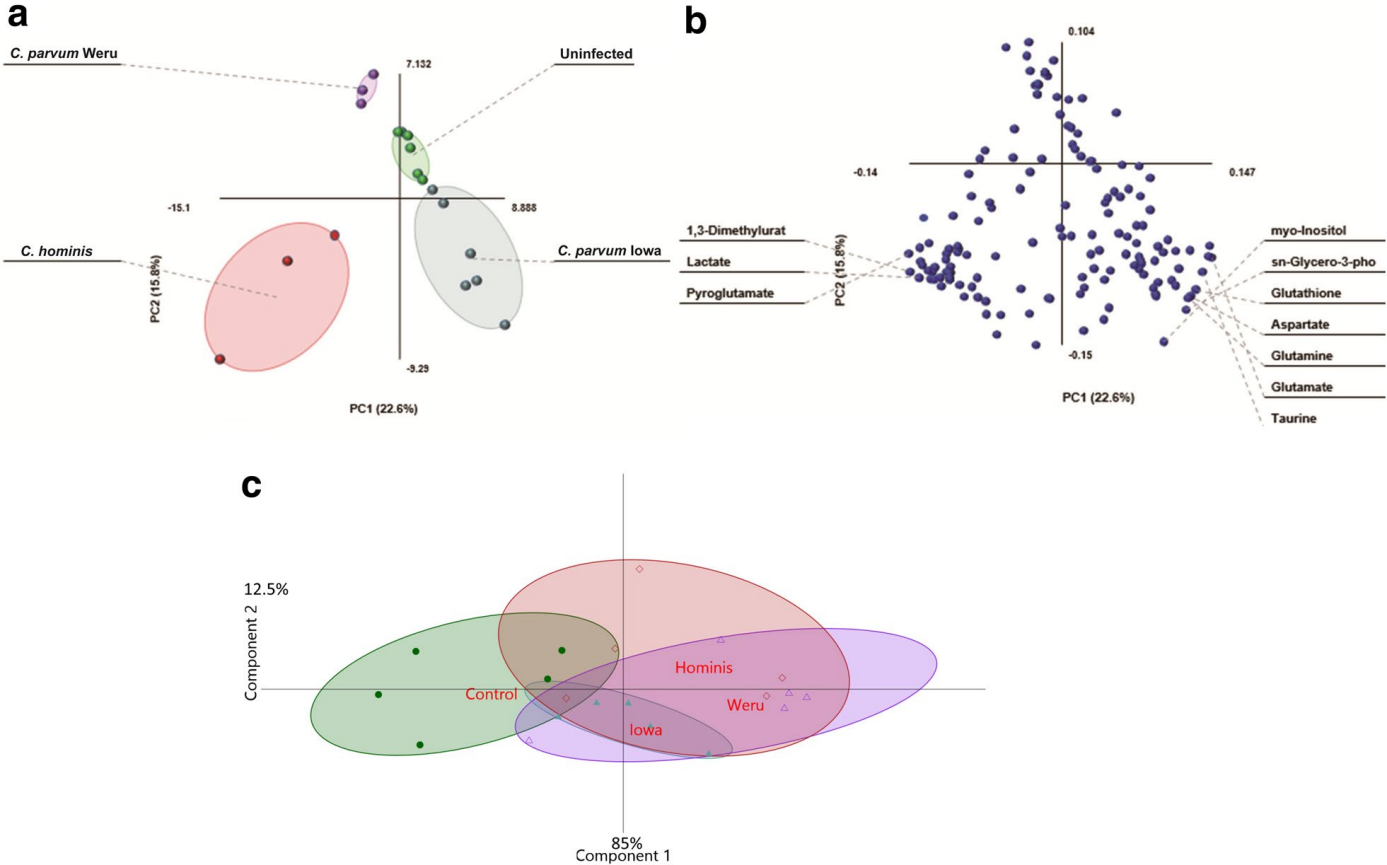
PLS-DA and loading plot of COLO-680N—infected cells NMR results. **a** PLS-DA statistical analysis of the information provided by the Chenomx screening produced clear groupings, separating the controls (green), *C. parvum* Iowa II infections (blue), *C. parvum* Weru infections (purple) and *C. hominis* infections (red), 7 days post infection. As the grouping areas do no overlap the separation between the infection conditions again indicates that metabolome differences can be at least in part explained by different *Cryptosporidium* strains/species. **b** The loading biplot of the PLS-DA analysis shows lactate as a significant contributor to variation, in addition to taurine and myo-inositol among others. **c** PLS-DA statistical analysis of the information provided by the Chenomx screening using additional samples, also produced well-defined groupings, separating the controls (green), *C. parvum* Iowa II infections (blue), *C. parvum* Weru infections (purple) and *C. hominis* infections (red)

Extrapolated NMR data from COLO-680N (n = 38, *C. parvum* Iowa = 12, control = 12, *C. hominis *= 7, *C. parvum* Weru = 7) metabolite extractions, demonstrated clear differences between the metabolomes of each individual strain/species of *Cryptosporidium* infection (Fig. 1a). Differences could be observed between creatine, creatine phosphate, taurine and lactate spectra before the application of any analytical methods (Fig. 1b–d). Analysis using the Chenomx NMR Suite version 8.2 [35, 36] produced a list of 161 total compounds of varying concentrations across samples (Additional file 1: Figure S1). The partial least squares discriminant analysis (PLS-DA) generated, produced ample separation of the *Cryptosporidium*-infected and uninfected cultures in multiple experiments (Fig. 2a, c). Furthermore, the separation of the individual infection groups suggests that differences between both *Cryptosporidium* species and within individual strains of *C. parvum*, may elicit different metabolic responses in cell cultures. It is important to note that all data points obtained from the 38 individual samples were input into the calculations, as any outliers should be visible via the resulting PLS-DA plots and pre-emptive pruning of the data was deemed to be unreliable given the lack of pre-existing information on what should/shouldn’t be expected. The loading scores plot of the PLS-DA showed several compounds contributed heavily to the separations between groups, such as lactate, several fatty acid derivatives and taurine (Fig. 2b).

All metabolites identified in this manner were input into an online tool (MetaboAnalyst 3.0) producing a graph detailing which metabolic pathways were influenced by infection (Additional file 2: Figure S2) [37]. This approach identified several pathways, including the biosynthesis of various amino acids, as well as ketones and CoA (Additional file 2: Figure S2b–f). Within these pathways, metabolites were highlighted that were identified via the PLS-DA as contributing reliably towards differences between groups. Full compound names are available in Additional file 3: Figure S3.

### Mice faecal sample extractions

**Fig. 3:**
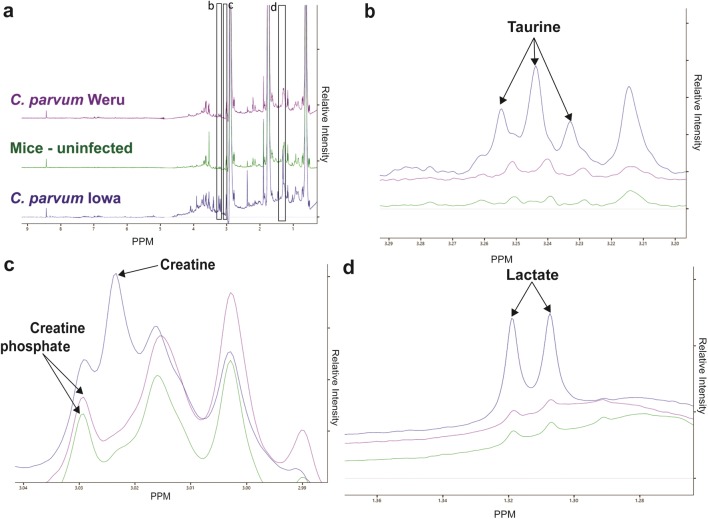
NMR Spectra of mice models of infection. **a** Stacked NMR Spectra produced from faecal samples of the control mice (green), or either the *C. parvum* Iowa II (blue) or *C. parvum* Weru (purple) groups, 10 days’ post infection. **b** Levels of taurine were substantially lower in the control or *C. parvum* Weru samples compared to *C. parvum* Iowa II. **c** Direct comparisons of the spectra revealed several clearly identifiably differences, including differences in creatine and creatine phosphate levels. **d** Lactate levels were also much higher in *C. parvum* Iowa II infected mice compared to the barely detectable levels in the control mice or *C. parvum* Weru infected groups

**Fig. 4:**
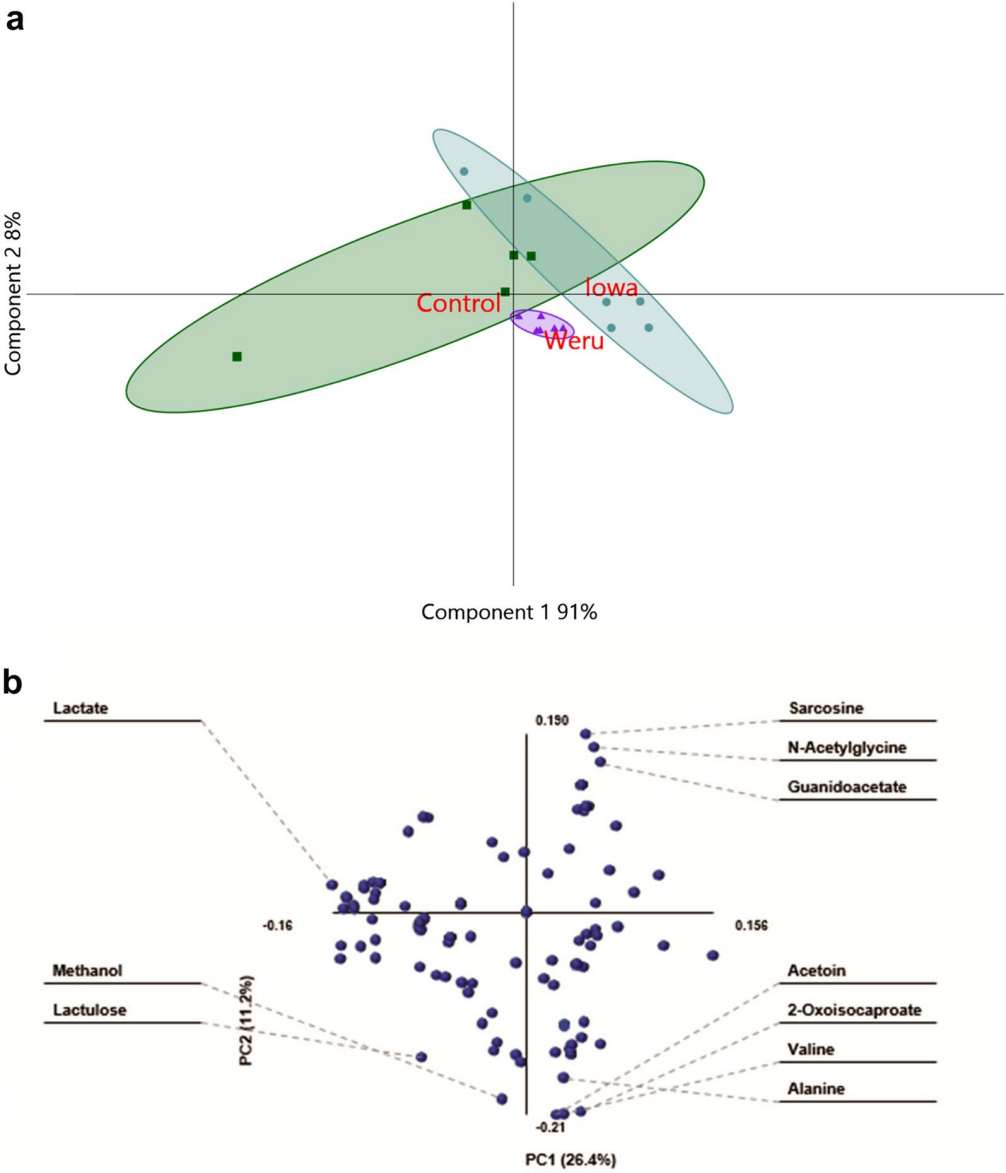
PLS-DA and loading plot of mice model NMR results. **a** PLS-DA statistical analysis of the information provided by the Chenomx screening produced clear groupings, separating the controls (green), *C. parvum* Iowa II infections (blue) and *C. parvum* Weru infections (purple), 10 days post infection. As the grouping areas, indicated by the areas highlighted, do overlap, it can be said that the separation between the infection conditions represent some differences in the metabolome, which correspond to the *C. parvum* strain. **b** The loading biplot of the PLS-DA analysis shows many of the compounds identified by Chenomx contributed towards the separation and groupings. Those on the outer most edges, for example alanine, sarcosine, lactate and lactulose, had some of the greatest influence on the amount of separation as determined by the PLS-DA

Faecal samples from infected and uninfected mice were smeared onto microscope slides and stained with an aniline-carbol-methyl violet method [38], allowing the detection of *C. parvum* oocysts and thus validation of successful infections (Additional file 4: Figure S4). Samples from both control and infected mice were taken at 10 days post infection, while monitoring and counting the number of oocysts. The spectra produced by the NMR showed clear distinctions between the infected and uninfected mice, as well as distinctions between the different strains of infections (Fig. 3a). Though 18 individual experiments were used to produce this data, the validity and reliability of each was confirmed by performing a further nine technical replicate NMR scans. Several metabolites were readily distinguishable prior to the metabolomics analyses, including indicators of phosphorylation; taurine (Fig. 3b), creatine and creatine phosphate (Fig. 3c) and lactate (Fig. 3d). Processing the data from the mice guts (n = 18, six per infection) via the Chenomx NMR Suite version 8.2 platform produced a list of 151 compounds that were extrapolated from the spectra (Additional file 5: Figure S5). Statistical analysis of the data, with freely available Microsoft Excel Add-in “multi-base 2015”, by PLS-DA determined some separation of the three conditions, (uninfected control, *C. parvum* Iowa II and *C. parvum* Weru infections), whilst maintaining group cohesion (Fig. 4a). The loading values of the variable compound contributions (Fig. 4b), suggest certain metabolites were more significant to the separation of the groups than others. The presence of l-alanine and valine, two common amino acids, agrees with the previous literature and 2-oxoisocaproate is a component of the valine/leucine/isoleucine biosynthetic pathways reports [27, 28].

MetaboAnalyst 3.0 based analysis of the metabolites proposed that several amino acid biosynthesis pathways could be altered during an infection, such as the glycine, valine and taurine pathways. In addition, the mice infections displayed possible changes to other metabolic pathways (Additional file 6: Figure S6a) as those pathways furthest from the x, y axis intercept, representing both the overall completeness of the pathways and number of contributing detected metabolites respectively. As with Additional file 2: Figure S2a–g, the pathways identified in the manner, and the compounds discovered by the NMR demonstrated that infections caused changes in at least the valine (Additional file 6: Figure S6c), glycine (Additional file 6: Figure S6d) and taurine amino (Additional file 6: Figure S6e) acid biosynthetic pathways, in addition to several sugar pathways (Additional file 6: Figure S6b, f, g). As before, full compound names are available in Additional file 3: Figure S3.

### Comparison of mice faecal and COLO-680N metabolome changes

**Fig. 5:**
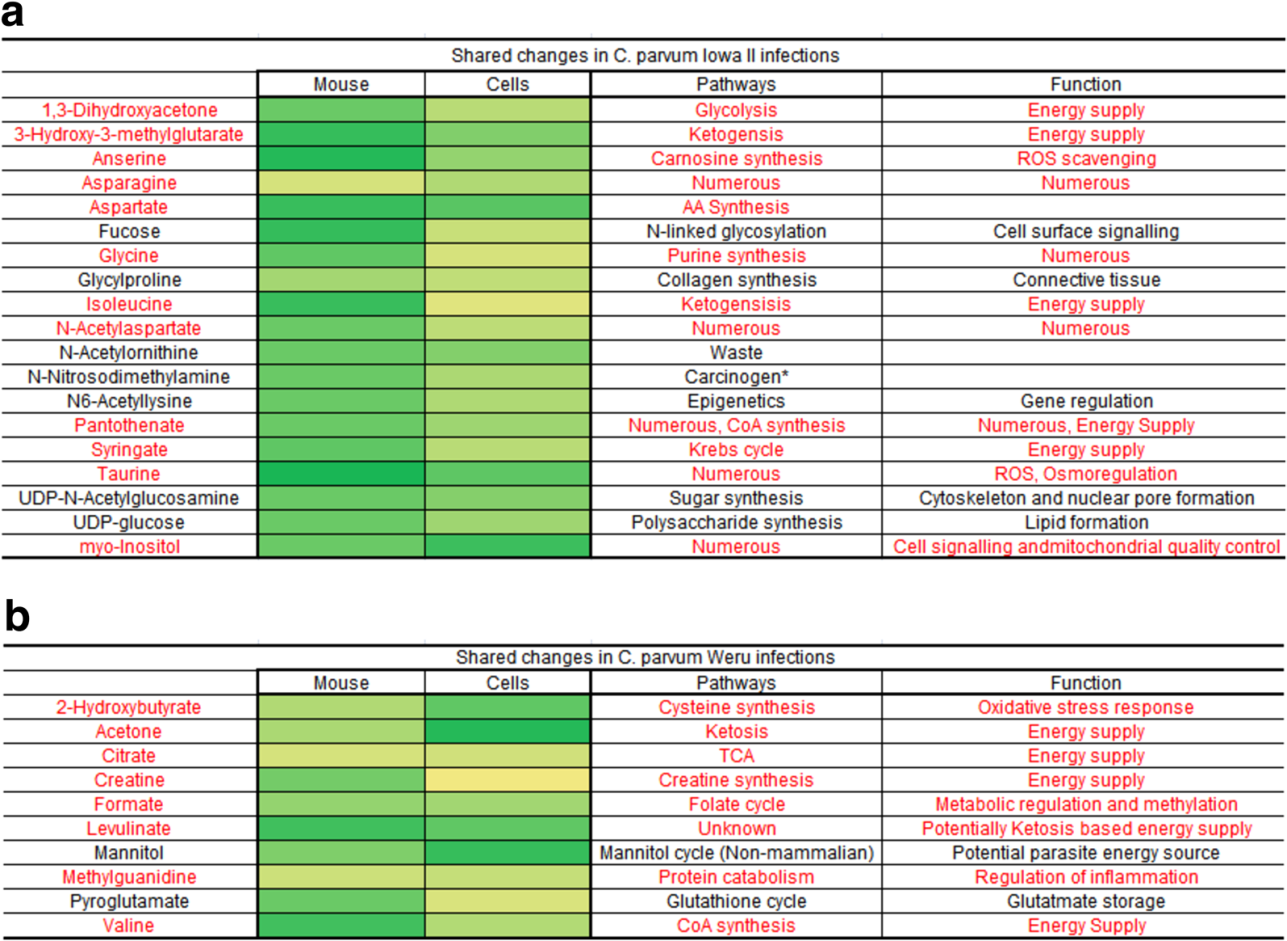
Shared changes in metabolite levels between both cell cultures and mice. Those metabolites which showed a reliable contribution towards group separation (determined by positive PLS-DA values, displayed as green shaded cells in Additional file 1: Figure S1 and Additional file 5: S5) in both mice and cell culture experiments were recorded and their functions assigned. Those metabolites with direct or indirect involvement with mitochondria are labelled in red. The analysis was conducted for both the *C. parvum* Iowa II (**a**) and *C. parvum* Weru (**b**) infection experiments. *N-nitrosodimethylamine is a known carcinogen and not naturally produced by any known human or mammalian cell line or any member of the cryptosporidia and may represent either a product of the gut microbiota, contamination or un-characterised spectra peak

MetaboAnalyst data from Additional file 2: Figure S2 and Additional file 6: Figure S6, demonstrate that a number of altered pathways are shared between the mice (faecal) and cell culture metabolites, particularly taurine and amino acid metabolic pathways. Glycine synthesis was also shown to be affected to a large degree. Comparing the data from the mouse and cell culture responses directly revealed many metabolite levels responded similarly to infection regardless of host (Fig. 5).

## Discussion

Previous studies recently demonstrated the successful long-term propagation of *C. parvum* in COLO-680N cell culture [22]. The ability of the cell culture to maintain the parasite for up to 8 weeks [22, 23] along with the presence of organelles around the parasite (e.g. feeder organelle) [21], implied a metabolic association between the parasite and the host. To investigate this, we have used ^1^H NMR to explore the metabolomics of the infection.

Solution-state ^1^H NMR offers a practical approach to metabolomics that is especially useful where sample volume sizes are particularly small [32, 33, 39]. Although GC–MS holds an advantage for detecting low-levels of metabolites with unique mass signatures, to determine the change in metabolite quantities, NMR provides a viable alternative [29–34]. Initial analysis of our data showed a clear distinction between the metabolic fingerprints of infected and uninfected samples, even between infections of different strains of the parasite to some extent; with PLS-DA producing distinct groups of metabolite profiles, correlating to uninfected and infected samples (Fig. 2a, c). This may in-part be explained as the manifestation of the biochemical differences between the species which contribute to their observed species specificity.

Of importance is the degree to which these results, both from the in vitro and in vivo, agree with the previous literature. As a monolayered, simplistic culturing system, it should not be expected that the results would be a perfect mimicry of an in vivo experiment, although their similarity to other studies should indicate that the COLO-680N culture is a pragmatically sufficient model for infection in more generalised studies such as this one. Our study also demonstrates that metabolic compounds l-alanine, isoleucine and succinic acid (succinate) were detected as contributors to the variance between the sample conditions that indicated infection. Moreover, even though valine was not detected in the uninfected controls, it was visible in the infected samples and in agreement with previous studies [27, 28].

The MetaboAnalyst data revealed several pathways were potentially influenced by infection, including several that showed changes in both the mice and cell culture experiments, such as amino acid and CoA biosynthesis. Support for these findings is observed via the biosynthesis pathways for alanine and glycine that were highlighted previously in GC-MS studies as being potentially influenced by infection [27, 28]. It is, however, important to note that these findings are based entirely on the presence/absence or relative changes in abundance of the noted metabolites and therefore cannot account for their specific origin. For example, it is not possible to distinguish between an increase in metabolite level due to damage of a pathway, compared to deliberate upregulation of a pathway, or even to the alterations of the gut microbiota. We hope to mitigate this by comparing the previous standards of these experiments, the mouse faecal samples, to the cell culture results.

As a parasite, *Cryptosporidium* is dependent on host derived biosynthetic pathways for survival. For example, *C. parvum* is incapable of producing most amino acids de-novo, instead relying heavily on the import of host metabolites via active channelling [40]. The biosynthetic pathway for glycine, threonine and serine was upregulated, in both cell culture and animal experimentations, with particularly high levels of glycine detected. Both *C. parvum* and *C. hominis* are incapable of manufacturing these amino acids de novo, instead relying on scavenging host serine and glycine, utilising serine and glycine hydroxymethyltransferases to convert one to the other when needed [40, 41]. The reliance on host amino acids could provide a novel method for combating the infection, based upon previous studies that identified other amino acid metabolic chains as potential targets [41, 42]. For example, glycine reuptake inhibitors (GRIs) that are often used in treating schizophrenia, could be utilised to partially starve the parasite of the metabolite.

In addition to the amino acid biosynthesis pathways, it is also apparent that taurine synthesis is also implicated in the metabolic profile of the disease as shown in the presented analyses; taurine has frequently been used in the past as an agent for inducing excystation for in vitro cultures as sodium taurocholate [43–46]. In the host, taurine has several roles, those relevant to the cell types involved include: cell membrane integrity, osmoregulation and adipose tissue regulation. Perhaps most notably, however, is the role taurine plays as a pH regulator in the host mitochondria. The addition of taurine is another piece of evidence indicating host-mitochondria are somehow directly involved in the progress of infection. Previous metabolomic studies of faecal samples from *Cryptosporidium*-infected patients revealed increased taurine concentrations, which was explained away as characteristic of a decline in gut absorption as a result villi malformation [47, 48]. However, malabsorption is not an applicable explanation in the infected COLO-680N cell cultures, wherein there is no external source of the metabolite and thus is likely correlated to the infection metabolome. Increases were also observed in the abundance of adenosine derivatives (AMP, ADP and ATP); all showing an increased abundance in infected cells and mice in *C. parvum* Iowa II infections, along with a similar increase in creatine levels in *C. parvum* Weru infections. This further implicates the role of host mitochondria in the context of infection as each species and strain of parasite lacks creatine kinase, the only alternative source therefore being host creatine kinase which is often found in close association with mitochondria. Levels of pyruvate in *C. hominis* cell cultures and pantothenate in *C. parvum* Iowa II mouse infections suggest interactions with oxidative phosphorylation. This is of interest as the *C. parvum* genome contains a sequence for a potential pantothenate scavenging protein [49]. Moreover, the further increase in lactate levels detected in *C. hominis* cell cultures and *C. parvum* Iowa II mouse infected samples, compared to the controls, indicate a strong contribution from anaerobic pathways most likely from the host. This suggests that more ATP is being produced than the oxidative capacity of the host mitochondria alone can maintain, producing a net increase in lactate as the oxygen debt increases. This suggests either an atypical drain of cellular ATP or a decrease in host cell aerobic capacity. Similar observations have been made in other intracellular parasites, including the microsporidian *Encephalitozoon cuniculi*, in which the organism acquired specialised transporters to overcome its needs for ATP [50].

The findings above suggest that *C. parvum* and *C. hominis* infections are directly or indirectly inducing an increase in host mitochondrial activity. If factual, this would result in many oxygen free radicals being produced by the metabolic machinery. Consequently, cell(s) would respond with a matching increase in the synthesis of antioxidants such as taurine, which also sees increases during infection [51–53]. However, there also exists non-related rationale for the detected increase in taurine, for example as a diuretic, which should not be a surprise in cryptosporidiosis, which is characterised by excessive water loss. This role sees taurine maintaining levels of the ionised forms of magnesium and potassium within the cell, producing a diuretic effect that may contribute towards the characteristic water-loss [48, 54–56]. Furthermore, it has been found that taurine levels influence production of short chained fatty acid, another aspect of host biology theorised to be scavenged by *C. parvum* and *C. hominis* [56–58]. Previous studies which have identified a rise in taurine levels in cryptosporidiosis patients’ stool, have dismissed the event as simply the result of the guts decrease in absorptive qualities. The presence of increased taurine in the in vitro samples, which lack external sources that could be responsible for a build-up, would appear to dispute this conclusion. It is our interpretation, therefore, that the intracellular role of taurine in this disease has been overlooked and that the pathophysiology of this disease is more complicated than currently understood, extending beyond villi degradation.

Lastly, these results provide a potential for determining infections via a possible comparative ^1^H NMR of patient and reference biopsies. This would offer an alternative approach in the medical field, where current methods of diagnosis are reliant on multiple, separate, techniques to achieve the same result as NMR, with infections detected by laborious and often inaccurate microscopy in tandem with strain typing dependant on a successful PCR.

## Conclusion

In conclusion, we have demonstrated for the first time that the use of ^1^H NMR in the context of both medical and scientific applications is indispensable in the fight against cryptosporidiosis. With the application of a more user-friendly and reproducible approach of metabolomics, through the ^1^H NMR methodology described in this paper, it will now be easier for the *Cryptosporidium* community to further explore the remaining aspects of the disease metabolome in patients’ samples.

## Methods

### Cryptosporidium

Three isolates of *Cryptosporidium* were used in this study. The reference strain *C. parvum* Iowa II was obtained from Bunch Grass Farm in the United States, isolated from infected calves. The human isolate *Cryptosporidium parvum* Weru strain was originally isolated from an infected human patient and subsequently maintained by passing through SCID mice and supplied courtesy of Prof. Martin Kváč of the Institute of Parasitology Biology Centre CAS, Czech Republic. The final isolate used was the human isolate of *C. hominis*, supplied courtesy of Prof. Rachel Chalmers from the *Cryptosporidium* Reference Unit, Singleton Hospital of NHS Wales.

### Cell culture

75 cm^2^ monolayers of COLO-680N were infected and maintained as per the protocols outlined previously [22], using all three isolates of *Cryptosporidium*. In brief, for a typical infection, 4 × 10^6^ oocysts were used to infect 75 cm^2^ cell culture flasks at between 70 and 80% confluency (2 × 10^6^ cells) giving a multiplicity of infection (MOI) of approximately 2. Infected cells were incubated for 7 days and monitored daily for their infectivity [22, 23]. Prior to sample collection and metabolite extraction, the level of infection was monitored using Sporo-glo live staining under fluoresce microscopy [22, 24]. A control group was also established, following the same protocols as the infections, absent oocysts. Two separate experiments were executed using a minimum of five flasks per sample condition.

### Animals and infection

Pregnant female BALB/c mice (Charles River, Germany) were housed in plastic cages with sterilized wood-chip bedding situated in IVC Air Handling Solutions (Techniplast, Italy) with high-efficiency particulate air (HEPA) filters and supplied with sterilized food and water ad libitum. For this study, 7 day old BALB/c mice from the same mother, habiting the same cage were infected at the Institute of Parasitology, Biology Centre CAS using pre-established protocols detailed in Meloni and Thompson, totalling five mice per condition [59]. Three separate conditions, totalling six animals each, were used, infecting with 100,000 oocysts of *C. parvum* Iowa II resuspended in 50 µl of PBS, 100,000 oocysts of the *C. parvum* Weru isolate resuspended in 50 μl of PBS or a PBS control (50 μl), given by oral gavage. The groups were kept physically separated and never allowed to interact. Infection was monitored and oocyst production was quantified from day-1 post-infection by aniline-carbol-methyl violet staining of faecal smears [60], RIDA^®^QUICK *Cryptosporidium*, supplied by R-Biopharm. At 10-days post-infection, the mice were euthanized by cervical dislocation and decapitation. Samples of the ileum were dissected from the mice, measured to the same size to ensure reproducibility. *Cryptosporidium hominis* was not used in the mice infection experiments as it has previously shown that this species cannot infect these animals [61].

### Sample preparation for NMR

The following protocol was adapted from published and well-established metabolic extraction methods used for NMR-based untargeted analysis of cell extracts [62–65]. Samples collected from the mouse experiments were retrieved from the contents of the ileum and surrounding intestinal structure. A section of ileum approximately 5 mm in length was removed from the euthanised mouse by scalpel. A syringe containing 3 ml of 100% ethanol at room temperature was the inserted into the removed ileum and the ethanol pushed through the ileum over a petri dish. The sample was then collected via pipette and stored in three 1.5 ml tubes in 1 ml aliquots.

Collected samples were then centrifuged for 3 min at 10,000×*g*, the supernatant discarded, and the pellet weights recorded. The samples were then suspended by vortex in 2 ml of 75% ethanol, pre-heated to 80 °C, to immediately inhibit subsequent metabolic reactions, then transferred to a new tube and an additional five ml of 75% ethanol added.

Two microlitre of 2 mm diameter glass beads were added to the samples and agitated by vortex for 30 s before incubating the samples for 3 min at 80 °C. The samples were vortexed for a further 30 s or until the sample was completely homogenised. Cell culture samples were collected by draining the media, adding 6 ml of ethanol at 80 °C to the culture flask and scraping the cells off the surface by cell scraper, transferring the mixture of lysed cells into 15 ml polyethylene tubes via a 10-ml serological pipette.

The samples were then transferred into 2 ml tubes, retaining the glass beads in 15 ml conical tubes. The beads were washed with an additional two ml of 80 °C, 75% ethanol and again the liquid was transferred into sterile 2 ml tubes, retaining the glass beads in the tube.

Cell debris and general detritus were separated from the metabolite samples by centrifugation at 16,000×*g* for 10 min at room temperature and the resulting supernatant transferred to new, sterile 2 ml microcentrifuge tubes and the remaining debris weighed for data normalisation. The samples were then dried via Rotorvac for 12 h or until completely desiccated, at 40 °C, suspended in 330 μl double distilled water and centrifuged at 2500×*g* for 10 min. The supernatants were recombined into ~ 1 ml aliquots per original sample in sterile 1.5 ml microcentrifuge tubes and frozen at − 20 °C until the day before NMR analysis. The sample tubes are subsequently placed into a freeze drier until completely desiccated, suspended in 1 ml of deuterium oxide (^2^H_2_O) and spiked with the sodium salt of the calibration and quantitation control compound: 3-(trimethylsilyl)-1-propanesulfonic acid (DSS), to a final concentration of 20 μM and a tested pH of 7.5.

### NMR protocol and analysis

Samples were analysed using a 4-channel Bruker Avance III 14.1 T NMR spectrometer (600 MHz ^1^H) equipped with a 5 mm QCI-F cryoprobe. For controls: six separate, uninfected 25 cm^2^ COLO-680N 100% confluent monolayer cultures were analysed in addition to three uninfected BALB/c mice. Infected samples consisted of six 25 cm^2^ COLO-680N 100% confluent monolayers in addition to three *c. parvum* Iowa II infected BALB/c and three *C. parvum* Weru infected BALB/c mice. One-dimension NMR datasets were acquired with a pulse repetition rate of 5 s over 128 scans, preceded by eight equilibrating dummy scans and suppression of the residual Deuterium Oxide solvent (HDO) resonance using presaturation. This was repeated 5 times per sample to ensure the reliability of the spectra produced. Processed NMR spectrographic datasets were produced by Topspin 3.2 and analysed using Chenomx NMR Suite version 8.2. Partial Least Squares Discriminant Analysis (PLS-DA) of the Chenomx data were generated with the freely available Microsoft Excel Add-in “multi-base 2015” by Numerical Dynamics, Japan (“Mutlibase for Microsoft Excel,” 2015) and “Past3.x” by Øyvind Hammer, Natural History Museum, University of Oslo. Pathway predictions were produced by the MetaboAnalyst 3.0 web tool, using a hypergeometric test and relative-betweeness centrality (measure of centrality in a graph based on shortest paths) against *Homo sapiens* and *Mus musculus* databases for the tissue culture and mouse models respectively [37]. However, the analysis methods do contain limitations, relying largely on human interpretation of the initial results. As such it is not possible to determine whether a result is erroneous or simply unexpected and be able to prove the decision was free of bias, we elected to include all the findings from the analysis in this paper. In this case, the limitation is also a result of database comprehensiveness, whilst standards can be used in the lab to determine the accuracy of the dataset, it cannot detect compounds it has not been trained to recognise. Furthermore, as a result it is possible that such compounds may be misidentified, caffeine for example is highly unlikely to be present in the sample but in the spirit of intellectual integrity the results have not be edited or altered in any way other than those required for easier reading. Furthermore, compounds have been labelled based on their most likely source (human metabolome for COLO-680N samples, mouse metabolome for faecal (gut microbiome) samples or *C. parvum* if not found in either of the previous), whilst some (such as acetyl ornithine) may serve a purpose beyond “waste product” in other organisms, in this paper we do not consider potential outside sources [37]. On the other hand, as is the case with all new technologies and techniques, these limitations can only shrink with repeated use as more data becomes available to fill in the gaps.

## Additional files

**Additional file 1: Figure S1.** COLO-680N Experiment Metabolites. All the metabolites identified by 1H NMR analysis in infected and uninfected cells were explored via PLS-DA statistical analysis and the resulting values of each individual metabolite recorded. The colour coded heat map represents the significance to which each individual metabolite contributed to the identity of the sample groups. Red indicates that a metabolite showed large amounts of variance *within* identically treated samples, yellow indicates that the amount of a metabolite varied little throughout all samples and green indicates that the metabolite was uniform within both control and infection groups but demonstrated a marked difference between them. Colour intensities were determined relatively from loading values, using the lowest negative value and highest positive value as the anchor points for red and green respectively, anchoring the mid-point yellow as 0.

**Additional file 2: Figure S2.** Metabolic pathways detected in cell cultures’ NMR samples. **a** Data analysed by MetaboAnalyst 3.0, utilising all compounds which displayed some degree of change as a result of infection, produced a graph of pathways most heavily impacted (x axis) and pathways containing the most amount of the given compounds (pathway impact: y-axis), with statistical significance of the predicted pathways increasing as the colour ranges from yellow (low) to red (high). Six pathways were chosen to be of particular interest by their position on the graph, with metabolites present in the experimental samples highlighted in red, including: glycine, serine and threonine metabolism (**b**), taurine and hypotaurine metabolism (**c**), Alanine, aspartate and glutamate metabolism (**d**), synthesis and degradation of ketones (**e**), pantothenate and CoA biosynthesis (**f**) and arginine and proline metabolism (**g**).

**Additional file 3: Figure S3.** Compound code key. KEGG ID to Compound name conversion table for use with Additional file 2: Figure S2 and Additional file 5: Figure S5.

**Additional file 4: Figure S4.** Staining of *Cryptosporidium* in faecal samples. Aniline-carbol-methyl violet stain of a faecal smear taken from a mouse in the infection group. The abundant presence of *Cryptosporidium* oocysts (arrows) indicates that the infection has been successful; and that the animal is producing oocysts. These samples were acquired at 7 days post-infection.

**Additional file 5: Figure S5.** Mice Experiment Metabolites. All the metabolites identified by ^1^H NMR analysis in infected and uninfected mice were explored via PLS-DA statistical analysis, the Principal Component values for each metabolite were then recorded. Metabolites that contributed towards variation *within* groupings are coded towards the red, whilst green represents metabolites that stayed relative unvaried within groups but demonstrated variation between groups and thus are of most interest. Yellow represents a general lack of variation between or within groups.

**Additional file 6: Figure S6.** Metabolic pathways detected in mouse model NMR samples. **a** Data analysed by MetaboAnalyst 3.0, utilising all compounds which displayed some degree of change as a result of infection, produced a graph of pathways most heavily impacted (x axis) and pathways containing the most amount of the given compounds (pathway impact: y-axis), with statistical significance of the predicted pathways increasing as the colour ranges from yellow (low) to red (high). Six pathways were chosen to be of particular interest by their position on the graph, with metabolites present in the experimental samples highlighted in red, including: **b** pentose and glucuronate interconversions, valine, **c** valine, leucine and isoleucine biosynthesis, **d** glycine serine and threonine metabolism, **e** taurine and hypotaurine metabolism, **f** galactose metabolism and **g** starch and sucrose metabolism.

## Abbreviations

NMR: nuclear magnetic resonance
DSS: 3-(trimethylsilyl)-1-propanesulfonic acid, sodium salt
PLS-DA: principal component analysis
PLS-DA: partial least squares discriminant analysis
UV: ultraviolet
HIV: human immunodeficiency virus
GC–MS: gas chromatography–mass spectrometry
HDO: deuterium oxide
PCR: polymerase chain reaction
PBS: phosphate-buffered saline
EM: electron microscopy
SCID: severe combined immunodeficiency disease
ATP: adenosine triphosphate
AMP: adenosine monophosphate
ADP: adenosine diphosphate
CoA: coenzyme A
GRIs: glycine reuptake inhibitors
