# Supplementary figures and images for: NMR metabolomics reveals effects of Cryptosporidium infections on host cell metabolome
Source: Gut Pathog. 2019 Apr 3;11:13. doi: 10.1186/s13099-019-0293-x (PMC6446323; doi:10.1186/s13099-019-0293-x)

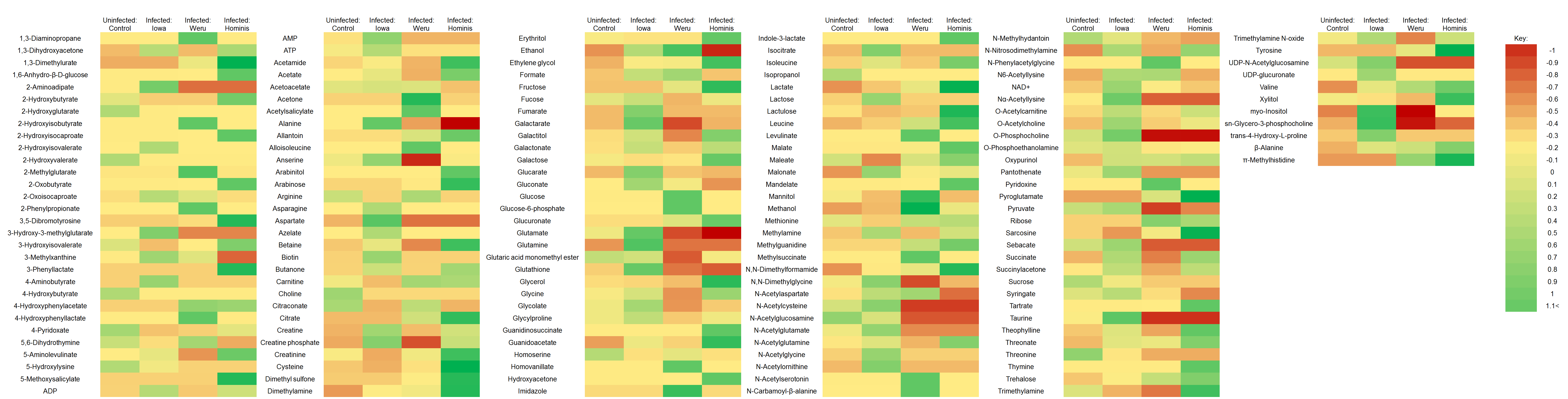

Supplement: Supplementary file 1 — Additional file 1: Figure S1. COLO-680N Experiment Metabolites. All the metabolites identified by 1H NMR analysis in infected and uninfected cells were explored via PLS-DA statistical analysis and the resulting values of each individual metabolite recorded. The colour coded heat map represents the significance to which each individual metabolite contributed to the identity of the sample groups. Red indicates that a metabolite showed large amounts of variance within identically treated samples, yellow indicates that the amount of a metabolite varied little throughout all samples and green indicates that the metabolite was uniform within both control and infection groups but demonstrated a marked difference between them. Colour intensities were determined relatively from loading values, using the lowest negative value and highest positive value as the anchor points for red and green respectively, anchoring the mid-point yellow as 0. [file 13099_2019_293_MOESM1_ESM.tif]

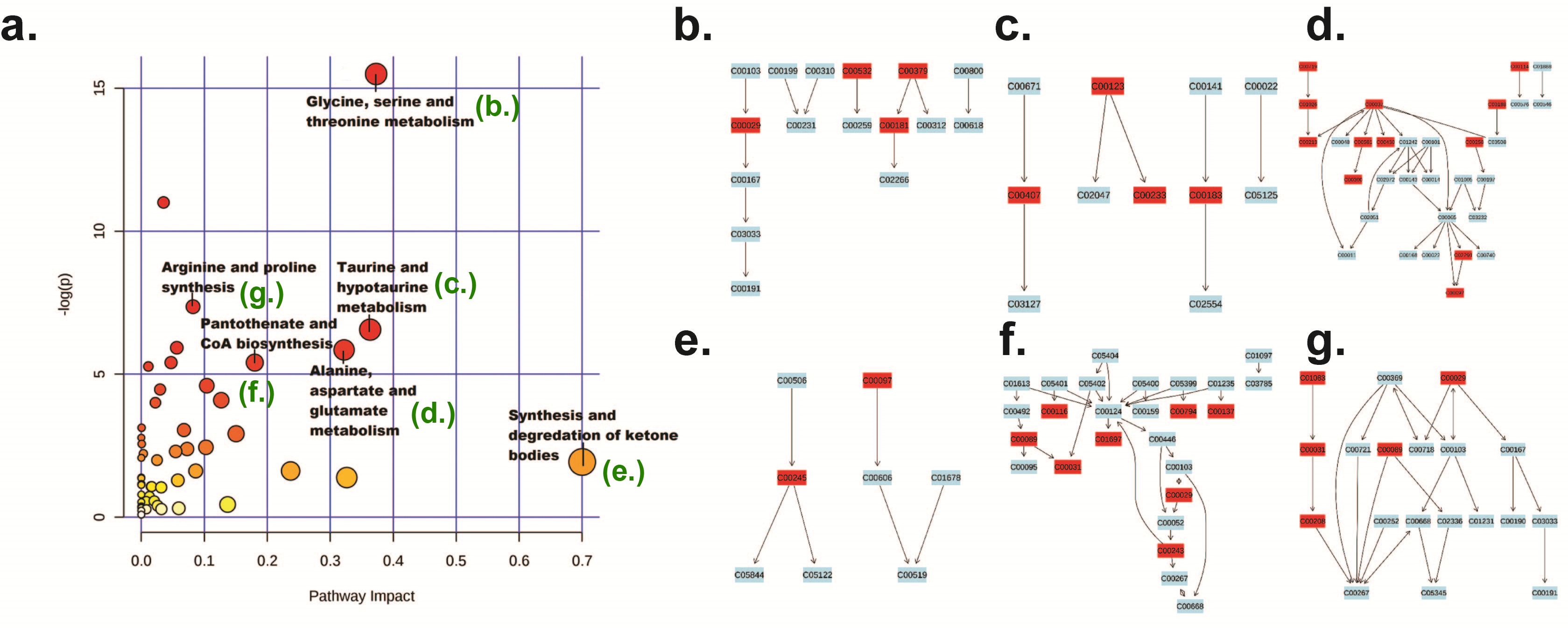

Supplement: Supplementary file 2 — Additional file 2: Figure S2. Metabolic pathways detected in cell cultures’ NMR samples. a Data analysed by MetaboAnalyst 3.0, utilising all compounds which displayed some degree of change as a result of infection, produced a graph of pathways most heavily impacted (x axis) and pathways containing the most amount of the given compounds (pathway impact: y-axis), with statistical significance of the predicted pathways increasing as the colour ranges from yellow (low) to red (high). Six pathways were chosen to be of particular interest by their position on the graph, with metabolites present in the experimental samples highlighted in red, including: glycine, serine and threonine metabolism (b), taurine and hypotaurine metabolism (c), Alanine, aspartate and glutamate metabolism (d), synthesis and degradation of ketones (e), pantothenate and CoA biosynthesis (f) and arginine and proline metabolism (g). [file 13099_2019_293_MOESM2_ESM.tif]

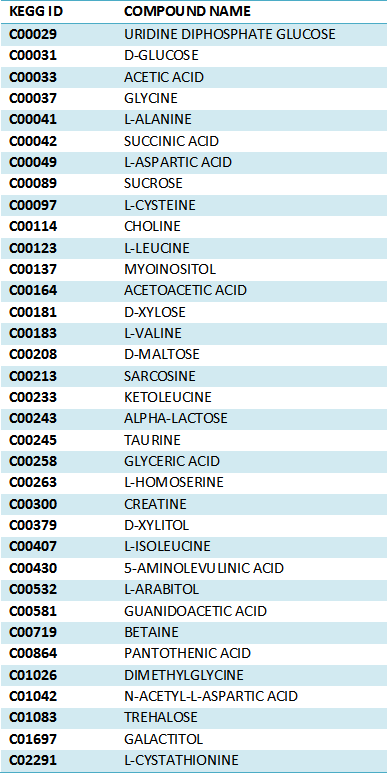

Supplement: Supplementary file 3 — Additional file 3: Figure S3. Compound code key. KEGG ID to Compound name conversion table for use with Additional file 2: Figure S2 and Additional file 5: Figure S5. [file 13099_2019_293_MOESM3_ESM.tif]

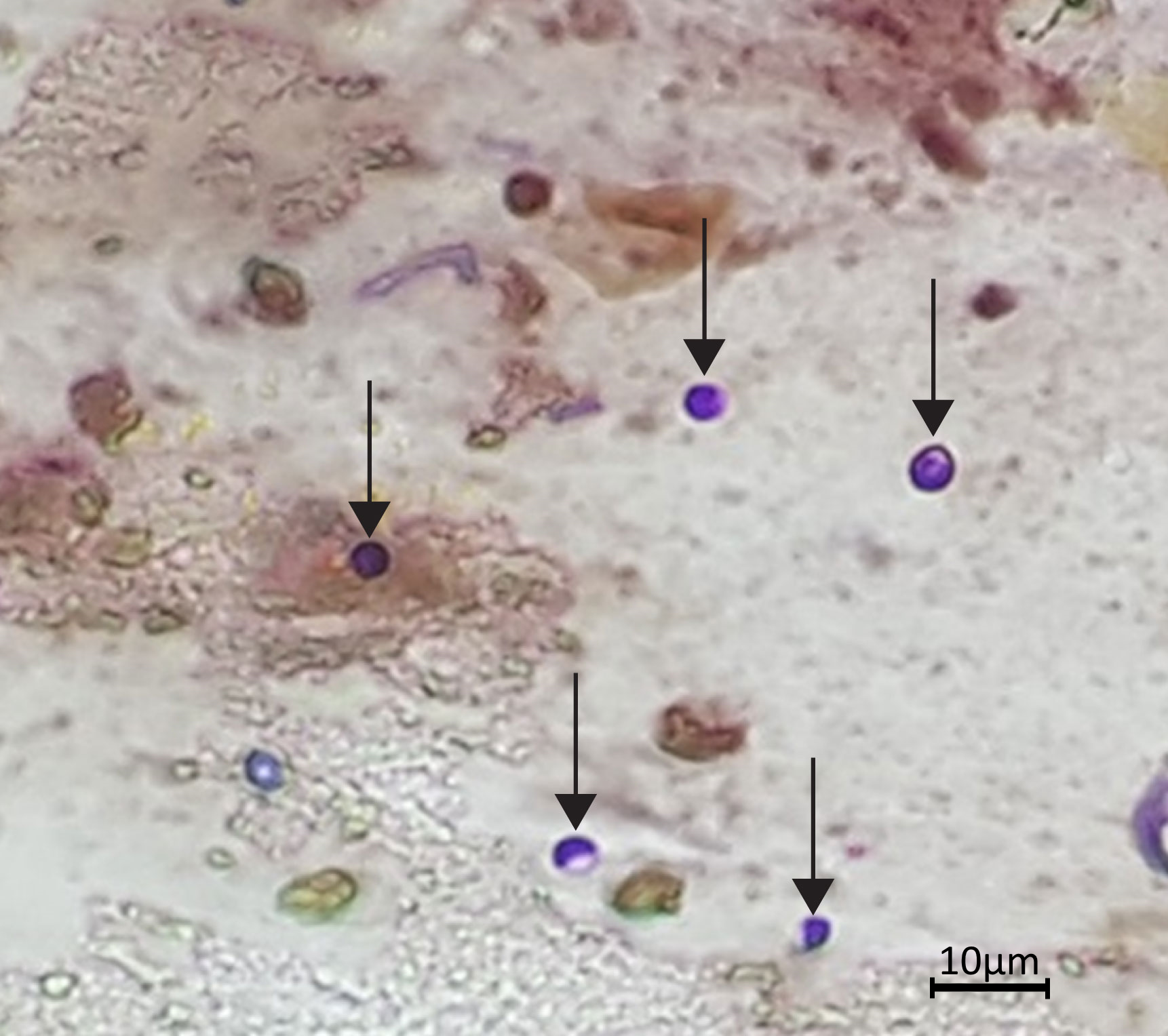

Supplement: Supplementary file 4 — Additional file 4: Figure S4. Staining of Cryptosporidium in faecal samples. Aniline-carbol-methyl violet stain of a faecal smear taken from a mouse in the infection group. The abundant presence of Cryptosporidium oocysts (arrows) indicates that the infection has been successful; and that the animal is producing oocysts. These samples were acquired at 7 days post-infection. [file 13099_2019_293_MOESM4_ESM.tif]

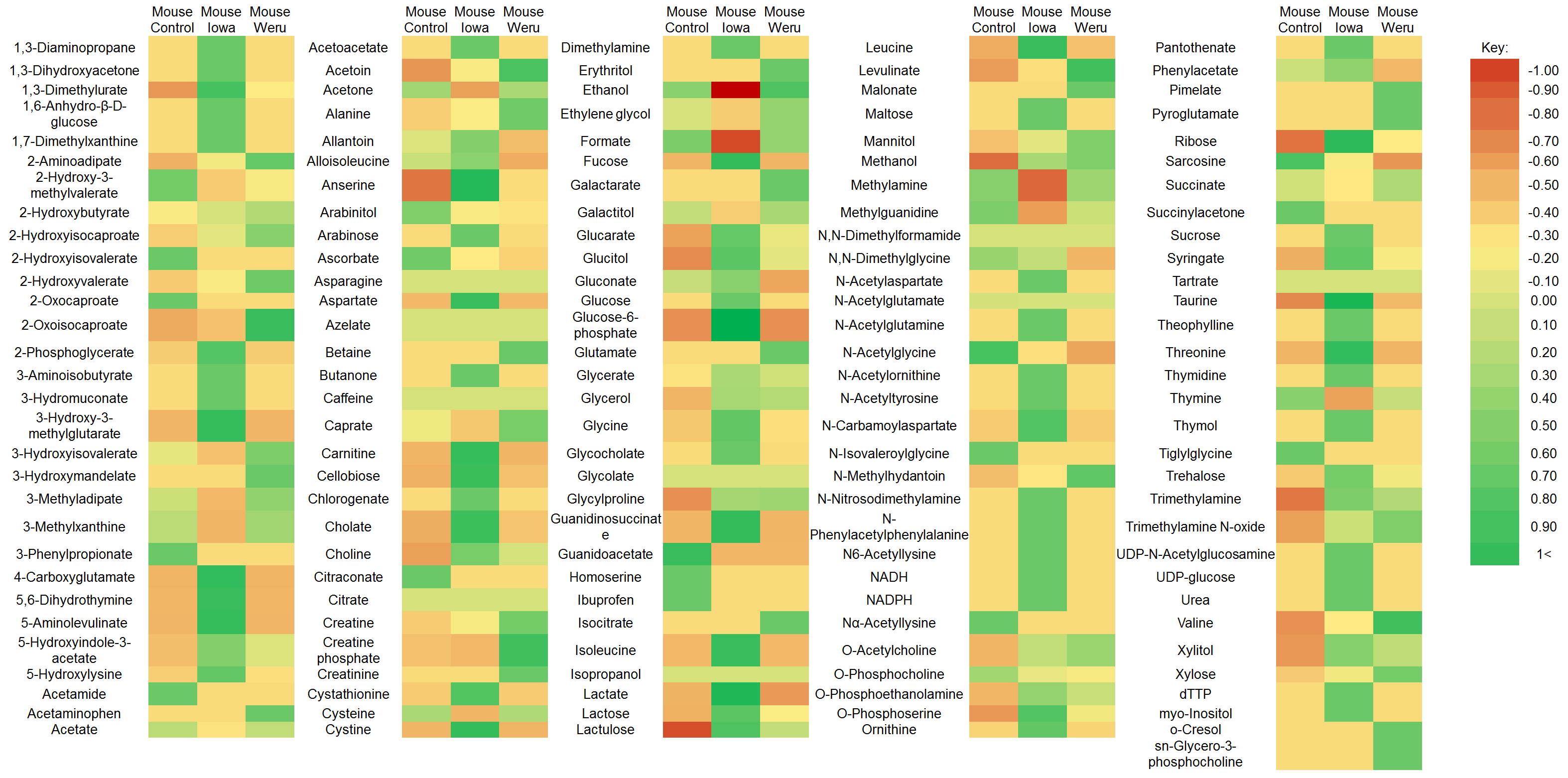

Supplement: Supplementary file 5 — Additional file 5: Figure S5. Mice Experiment Metabolites. All the metabolites identified by 1H NMR analysis in infected and uninfected mice were explored via PLS-DA statistical analysis, the Principal Component values for each metabolite were then recorded. Metabolites that contributed towards variation within groupings are coded towards the red, whilst green represents metabolites that stayed relative unvaried within groups but demonstrated variation between groups and thus are of most interest. Yellow represents a general lack of variation between or within groups. [file 13099_2019_293_MOESM5_ESM.tif]

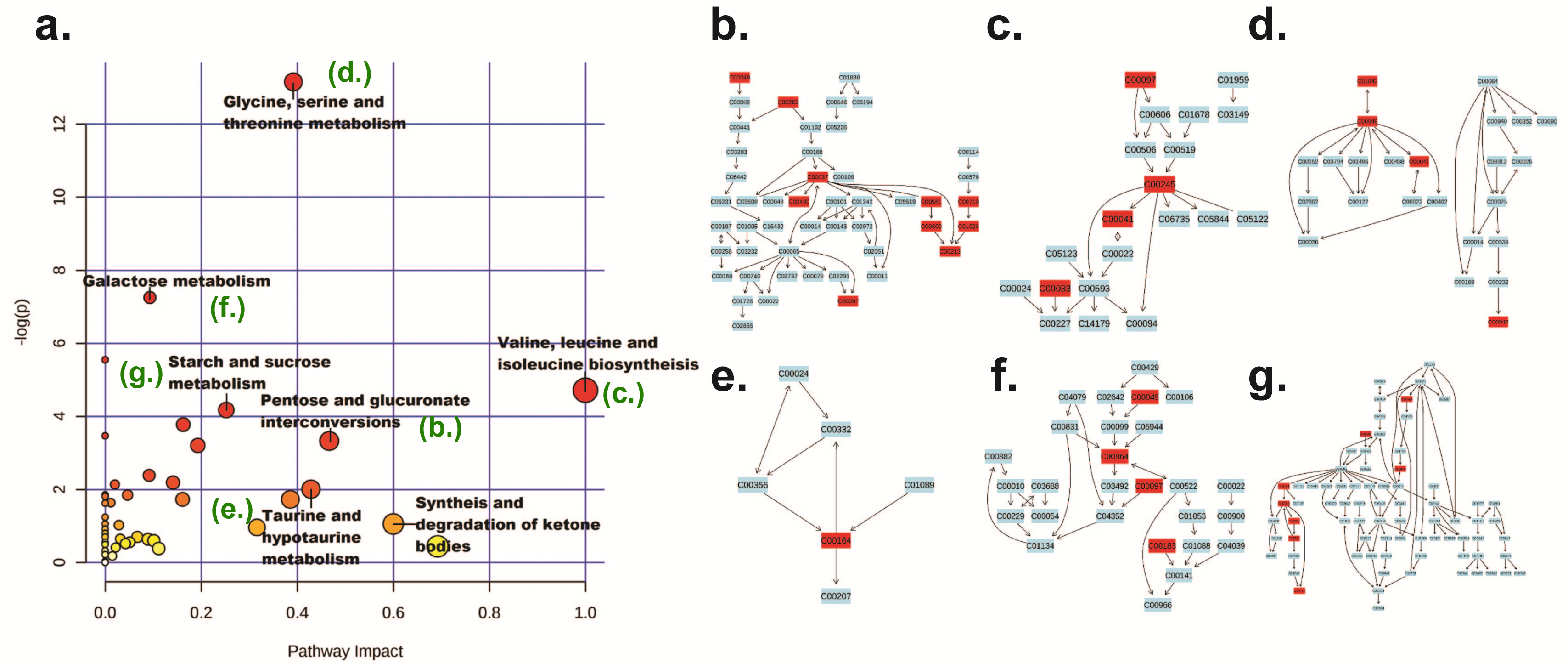

Supplement: Supplementary file 6 — Additional file 6: Figure S6. Metabolic pathways detected in mouse model NMR samples. a Data analysed by MetaboAnalyst 3.0, utilising all compounds which displayed some degree of change as a result of infection, produced a graph of pathways most heavily impacted (x axis) and pathways containing the most amount of the given compounds (pathway impact: y-axis), with statistical significance of the predicted pathways increasing as the colour ranges from yellow (low) to red (high). Six pathways were chosen to be of particular interest by their position on the graph, with metabolites present in the experimental samples highlighted in red, including: b pentose and glucuronate interconversions, valine, c valine, leucine and isoleucine biosynthesis, d glycine serine and threonine metabolism, e taurine and hypotaurine metabolism, f galactose metabolism and g starch and sucrose metabolism. [file 13099_2019_293_MOESM6_ESM.tif]
